# Sugarcane breeding: a fantastic past and promising future driven by technology and methods

**DOI:** 10.3389/fpls.2024.1375934

**Published:** 2024-03-08

**Authors:** Guilong Lu, Purui Liu, Qibin Wu, Shuzhen Zhang, Peifang Zhao, Yuebin Zhang, Youxiong Que

**Affiliations:** ^1^ National Key Laboratory of Tropical Crop Breeding, Institute of Tropical Bioscience and Biotechnology, Chinese Academy of Tropical Agricultural Sciences/Yunan Academy of Agricultural Sciences, Sanya/Kaiyuan, China; ^2^ College of Horticulture and Landscape Architecture, Henan Institute of Science and Technology, Xinxiang, China; ^3^ Key Laboratory of Sugarcane Biology and Genetic Breeding, Ministry of Agriculture and Rural Affairs, National Engineering Research Center for Sugarcane, College of Agriculture, Fujian Agriculture and Forestry University, Fuzhou, China

**Keywords:** sugarcane breeding, technology and methods, innovation, status, prospect

## Abstract

Sugarcane is the most important sugar and energy crop in the world. During sugarcane breeding, technology is the requirement and methods are the means. As we know, seed is the cornerstone of the development of the sugarcane industry. Over the past century, with the advancement of technology and the expansion of methods, sugarcane breeding has continued to improve, and sugarcane production has realized a leaping growth, providing a large amount of essential sugar and clean energy for the long-term mankind development, especially in the face of the future threats of world population explosion, reduction of available arable land, and various biotic and abiotic stresses. Moreover, due to narrow genetic foundation, serious varietal degradation, lack of breakthrough varieties, as well as long breeding cycle and low probability of gene polymerization, it is particularly important to realize the leapfrog development of sugarcane breeding by seizing the opportunity for the emerging Breeding 4.0, and making full use of modern biotechnology including but not limited to whole genome selection, transgene, gene editing, and synthetic biology, combined with information technology such as remote sensing and deep learning. In view of this, we focus on sugarcane breeding from the perspective of technology and methods, reviewing the main history, pointing out the current status and challenges, and providing a reasonable outlook on the prospects of smart breeding.

## Introduction

Sugarcane (*Saccharum* spp. hybrids) is one of the most promising industrial crops in the world (Kandel et al., 2018), originating in southern Asia in India, China, and New Guinea in the South Pacific, with a history of cultivation dating back about 10,000 years to New Guinea (Zhang et al., 2018). Initially, it was only grown in a few tropical countries, but with the recent breeding of superior varieties such as ‘POJ2878’, ‘NCo310’, ‘Co281’, ‘F134’ and ‘ROC22’, the cultivation has rapidly expanded to subtropical and even warm-temperate regions (Bhatt, 2020; Cursi et al., 2022a). Sugarcane is now cultivated in more than one hundred countries and regions, covering a total area of about 26 million hectares, with an annual production of 1.9 billion tons of fresh sugarcane, occupying nearly 80% sugar and 40% bioethanol of the world, with a total output value of $80 billion (FAO, 2021). In addition, sugarcane produces ethanol at an output-to-input ratio five times higher than that of maize (Waclawovsky et al., 2010) and can even be used for production of high-value chemicals (Rossi et al., 2021), and its by-products are highly exploitable for ethanol, animal feeds, cultivated substrates, and direct-fired power generation (Sindhu et al., 2016; Huang et al., 2020).

Variety is the “chip” of sugarcane industry. According to the International Society of Sugarcane Technologists (ISSCT), the scientific and technological contribution of sugarcane variety improvement is as high as 60% (Chen et al., 2011). Remarkably, all major producing countries in the world have taken the selection and breeding of new varieties and continuous upgrading as a solid guarantee to promote the steady development of sugarcane industry. However, sugarcane is an allopolyploid and aneuploid crop (2n=100-130, approximately 10 Gb) with a highly complex genetic background (Piperidis et al., 2010), and its hybrid progeny are widely segregated for traits (You et al., 2019), and the typical breeding cycle is as long as 10-15 years (Kandel et al., 2018). Notably, sugarcane is a tall-large (about 3-4 m tall), long-growing (about 10-14 months) perennial crop, and its phenotyping is a huge workload. Meanwhile, the development and utilization of wild germplasms are insufficient and breeding technology is relatively lagging behind in sugarcane breeding. Compared to crops such as rice, maize and soybean, the process of improving sugarcane varieties is extremely slow (Cursi et al., 2022b; Gonçalves et al., 2024). Currently, the main varieties in sugarcane production are mainly obtained through crossbreeding, and their progeny populations are huge, with as many as 1.0-1.2 million seedlings planted annually in mainland China alone (You et al., 2019).

As for global crop breeding, technological innovation has always promoted the leapfrog development. Accompanied by the evolution of natural species over thousands of years and the development of science and technology, agricultural breeding has gone through four stages, namely, primitive domestication and selection (Breeding 1.0), traditional or conventional breeding (Breeding 2.0), molecular breeding (Breeding 3.0), and smart breeding also known as molecular design breeding (Breeding 4.0), which is being transformed from theory to reality (Shen et al., 2022; Xu et al., 2022). Particularly noteworthy is that in 2018, the University of California realized asexual propagation of rice seeds for the first time, which helps to fix the hybrid advantage and lays a good theoretical foundation for the one-line method for breeding (Khanday et al., 2019). Recently, a group led by Lin Li from Huazhong Agricultural University in China published the first multi-omics integrative network map of maize in Nature Genetics, which provides a key to accurately decode the maize functional genome (Han et al., 2023). Researchers at the University of California have also developed “Cloning reprogramming and assembling tiled natural genomic DNA”, providing a simpler and more cost-effective way to build synthetic chromosomes (Coradini et al., 2023), which can be called the epochal masterpiece of plant Breeding 4.0. In that sense, it is time to discuss the technical difficulties and methodological strategies faced by sugarcane breeder, and to put forward the development ideas and research directions for sugarcane breeding in the future.

The world’s population is projected to exceed 9.7 billion by 2050, up from 7.95 billion in 2022 (United Nations, 2022), and the global demand for sugar and energy will increase further, all the while facing threats such as diminishing arable land resources, rising temperatures, and a highly unstable climate (Flack-Prain et al., 2021; Jaiswal et al., 2023; Olsson et al., 2023). As for sugarcane, huge economic losses are incurred every year due to the occurrence of diseases or pests such as smut, rust and borer (Cheavegatti-Gianotto et al., 2019; Gopi et al., 2024). Theoretically, it is encouraging to note that fresh stem yield of sugarcane can reach more than 380 t/ha (Waclawovsky et al., 2010), while the current yield is only about 70.6 t/ha and 83.2 t/ha, respectively (FAO, 2021), suggesting enormous room for improvement. In view of this, it is particularly necessary and urgent to seize the opportunity for breeding 4.0 era to fully develop the potential of sugarcane germplasm resources, and accelerate the breeding of breakthrough varieties through the use of modern technology. This perspective provides an overview of the achievements that have been made in the field of sugarcane breeding and elaborates the current status and future challenges from the aspects of technology and methods, with a special emphasis on smart breeding. We hope that it will accelerate the breeding process and technological innovations particularly in highly polyploid crops such as sugarcane.

## History of sugarcane breeding

### Breeding 1.0

**Figure 1:**
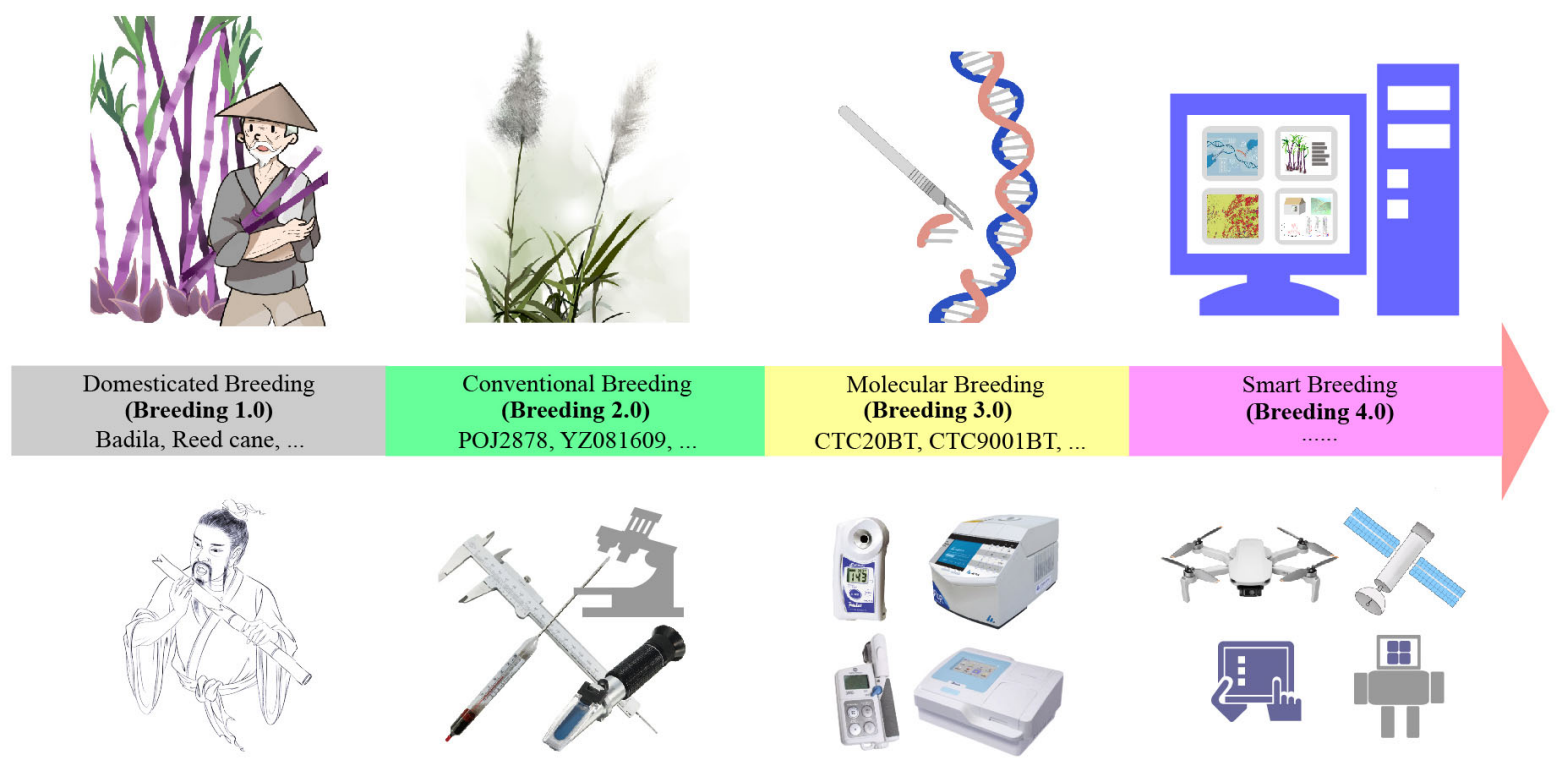
History and future perspective of sugarcane breeding.

In breeding 1.0 era, wild crops were domesticated and cultivated for food. Early farmers, although most probably did not understand the theory of genetic diversity, had already begun to use its value, consciously or unconsciously, to select plants by chance, choosing single plants that performed well in terms of yield or other traits in one season as “seeds” for the next season, and reproducing them over and over again (**Figure 1**). During this period, it was largely a matter of subjective judgement on the part of the farmer through visual observation of natural variation, and progress in crop improvement was very slow (Kuriakose et al., 2020; Zhang et al., 2023a). Regarding sugarcane, one hand, until the beginning of the 20th century, the raw material for sugar production in various countries, was only used in the original varieties such as bamboo cane, reed cane, Uba and Badila, or natural hybrids such as Greole and Bourbon. On the other hand, human beings have continuously developed sugar production technology for thousands of years (Chen et al., 2011; Wu et al., 2014). This has also laid the genetic resource base and technical enlightenment for the breeding of elite cultivars.

### Breeding 2.0

In the late 19th century and early 20th century, breeding has entered into the 2.0 era, which was characterized by cross-breeding strategies, quantitative genetics, and statistical approaches to select improved varieties according to Mendel’s plant genetic theory (Zhang et al., 2023a). In 1887-1888, Sotwedel F. in Java, and Harrison J. and Boyell J. R. in Barbados successively discovered natural sugarcane seedlings, which opened a new era of cross-breeding (Harrison, 1888; Griggs, 2011). In 1889-1890, Dutch sugarcane breeder Jeswiet J. pioneered the theory of “nobilization breeding”. He initiated a new way for sugarcane variety improvement, that is the interspecific hybridization between *S. officinarum* (female, noble cane) and *S. spontaneum* (male, wild cane) with few recurrent cycles of backcrossing between the first hybrids and the *S. officinarum* species. With this strategy, the first sugarcane complex ‘POJ2878’, which containing genes for high yield and high sugar in *S. officinarum* as well as genes for vigor, resistance to stress and disease in *S. spontaneum*, was created. By 1929, it had replaced almost all local cultivars, accounting for 95% of the total sugarcane planting area in Java, and had also become an elite cultivar and hybrid parent in many countries (Jeswiet, 1930; Widyasari et al., 2022). Subsequently, on the basis of work by Kobus J. D. and Barber C. A., Venkatraman T. S. bred ternary hybrids ‘Co213’, ‘Co281’ and ‘Co290’ containing the genetic characters from *S. officinarum*, *S. spontaneum* and *S. barberi* (Venkatraman, 1927; Ram et al., 2022), proving a new way to multiple the key regulating genes to improve sugarcane, and it was the second breakthrough in sugarcane breeding in the 20th century. Later, Mangelsdorf A. J. proposed the “Furnace hybridization”, which was the first to estimate the fitness of the parents, and a quadruple hybrid ‘H32-8560’ containing the kinship of *S. officinarum*, *S. spontaneum*, *S. barberi*, and *S. robustum* was bred from *S. robustum* (Mangelsdorf, 1956; Khan, 2022). Under this system, sugarcane breeders have also carried out their own cross-breeding programs and bred many famous sugarcane varieties, such as ‘CP’ series of Florida in the United States, ‘Q’ series of Queensland in Australia, ‘NCo’ series of Natal in South Africa, as well as ‘ROC’ series of Taiwan, ‘GuiTang (GT)’ series of Guangxi, ‘YueTang (YT)’ series Guangdong, and ‘YunZhe (YZ)’ series of Yunnan provinces in China (**Table 1**). Recently, Chinese sugarcane breeders used ‘YZ94-343’ (thick stalk) and ‘YT00-236’ (high sugar) to crossbreed ‘YZ081609’, a high-sugar variety with a peak sucrose value of 20.3% (Zhao et al., 2019; Zhang et al., 2024). They even used chemical or radiation mutagenesis to treat seeds or buds and sugarcane varieties with better characters were screened and selected (Chen et al., 2011; Penna et al., 2023).

**Table 1:** Sugarcane breeding achievements of major producing countries.

Country	Main objectives	Abbreviation of variety code	Typical varieties	Average production (t/ha) ^a^	Sugar production rate (%) ^b^	Disease resistance
Australia	high yield, high sugar, drought-resistant, disease-resistant, suitable for machine harvesting	KQ, Q	Q208, KQ228, Q200, Q183, Q232, Q138, Q226, MQ239, Q186, Q231, Q240	83.2	13.67%	brown rust, smut
Brazil	high yield, high sugar, disease-resistant, suitable for machine harvesting	RB, SP, CTC	RB86-7515, RB96-6928, SP81-3250, RB92-579, RB85-5156, RB85-5453	78.5	13.28%	smut
China	high yield, high sugar, drought-resistant, disease-resistant, suitable for machine harvesting	GT, LC, YT, YZ, ROC	GT42, GT44, LC05136, YZ081609, YZ0551, YT93-159	79.5	12.58% ^c^	smut, pokkah boeng, mosaic, brown rust
Cuba	high yield, high sugar, disease-resistant	C	C86-12, B7274, C323-68, C90-317, C86-156, C86-503, C86-531, C88-380 C90-530, C90-647, C89-250	43.5	6.12%	brown rust, yellow rust, mosaic
India	high yield, high sugar, drought-resistant, disease-resistant, saline-alkali resistance	Co, CoA, CoC, CoH, CoJ, CoLk, CoM, CoP, CoS, CoSe, CoSi, CoT, CoTL, CoV	Co0238, Co86032, CoA92082, CoM0265, CoS767, CoS8436, CoSe92423	74.32	10.20%	smut, red rot
Mexico	high yield, high sugar, disease-resistant	MEX	MEX69-290, MEX68-1345, MEX79-431, MEX91-662	67.87	11.63%	smut, brown rust, yellow rust
Pakistan	high yield, high sugar, disease-resistant	SPF, CPF, HSF, BF	BF-162, SPF-213, CPF-237, HSF-242	69.53	7.96%	smut
Philippines	high yield, high sugar, disease-resistant	VMC, PHIL	VMC86-550, PHIL2006-2289, PHIL2005-1763, PHIL2005-0483, PHIL8013	57.79	9.30%	smut, downy mildew
South Africa	high yield, high sugar, drought-resistant	NCo	NCo310	73.9	11.32%	smut
Thailand	high yield, high sugar, disease-resistant, strong perennial root	K, KK, LK, UT	KK3, LK92-11, K88-92, UT12	45.3	11.26%	smut, red rot
United States	high yield, high sugar, disease-resistant, suitable for machine harvesting, frost resistance	CP, HoCP, LCP, Ho, L	CP72-1210, CP89-2143, LCP85-384, HoCP96-540, L99-226	86.24	12.78%	brown rust, yellow rust, smut, ratoon stunting disease

a Production data from FAOSTAT;

b Sugar production rate from BRIC Agricultural Database;

c About sugar production rate: China calculates it based on the production of white granulated sugar, while other countries calculate it based on raw sugar.

### Breeding 3.0

Regarding breeding 3.0 era, breeders extensively utilized techniques such as molecular markers, transgene, gene editing and genomic selection to develop new varieties by selecting or aggregating the key regulating genes (Xu et al., 2022; Yadav et al., 2024). Molecular marker-assisted selection (MAS) breeding, which is reliable, efficient and not or rarely affected by the external environment, is the use of molecular markers closely linked to the target traits to assist breeding. Selection and planting of prevalent disease-resistant varieties is one of the most cost-effective ways to control sugarcane diseases and increase sugar content and cane yield (Aitken, 2022). However, so far, only the closely linked markers R12H16 and 9O20-F4 for resistance to the brown rust *Bru1* gene (Le Cunff et al., 2008) and the G1 marker for resistance to orange rust (Yang et al., 2018) have been used in sugarcane breeding. Recently, single nucleotide polymorphism (SNP) microarrays established on high-throughput sequencing began to be used for target trait localization, and quantitative trait locus (QTLs) related to yellow leaf (You et al., 2019), ratoon stunting (You et al., 2021), leaf blight (Wang et al., 2022a), and mosaic disease (Lu et al., 2023) resistance, as well as chlorophyll content (Lu et al., 2021), tillering and ratooning ability (Wang et al., 2023a) were obtained, nevertheless none of them have achieved subsequent progress in application.

To further reduce the cost while balancing the ability for statistical data analysis and process simplification, Giovannoni et al. (1991) and Michelmore et al. (1991) proposed a bulked segregation analysis (BSA) strategy. This approach is to use two parents with extreme differences in the target traits to construct a segregating population, and select the individuals with extreme phenotypic differences in their offspring to construct sequencing pools, respectively, which can effectively observe whether there is a significant difference in the allele frequency of polymorphic loci between the two populations, and then locate the markers associated with the target traits (Wang et al., 2023b). However, due to the complex genome of sugarcane, only a few traits related markers such as resistance to brown rust (Asnaghi et al., 2004), smut (Xu and Chen, 2004; Gao et al., 2022), leaf blight (Wang et al., 2022b), and brown stripe (Cheng et al., 2022) were obtained base on the BSA strategy.

Genome-wide association study (GWAS) or genomic selection (GS) is a new strategy for identifying genetic variation affecting a complex trait through comparative analysis at the genome-wide level (Tibbs Cortes et al., 2021). The former is intended to detect causal genomic regions that control the variation of quantitative traits, since statistically there are significant associations between genotype and phenotype. The latter is intended to explore all the available markers to fit genomic prediction models, which could replace the traditional phenotypic selection at a certain stage of a breeding program (Deomano et al., 2020; Yadav et al., 2020; Hayes et al., 2021; Islam et al., 2021; Mahadevaiah et al., 2021; Voss-Fels et al., 2021; Yadav et al., 2021; Islam et al., 2022; Sandhu et al., 2022; Islam et al., 2023). Natural populations are preferred for GWAS and GS due to their complex structure, obvious differences in genetic information, and the high number of rare variant loci. Compared to QTL localization based on genetic mapping, GWAS and GS have been reported relatively few in sugarcane. For example, GWAS was used for yield (Barreto et al., 2019; Zhang et al., 2023b), sugar content (Racedo et al., 2016; Fickett et al., 2019), fiber composition (Gouy et al., 2015; Yang et al., 2019a), drought tolerance (Wirojsirasak et al., 2023), and disease resistance such as yellow leaf (Débibakas et al., 2014) and orange rust (Yang et al., 2019b); and GS was used for fiber content, flowering (Hayes et al., 2021), yield and sugar (Deomano et al., 2020; Islam et al., 2022), ratooning ability (Islam et al., 2023), and rust resistance (Islam et al., 2021). It should be noted that GS has shown high prediction accuracies in practical breeding programs, and can be a promising tool for improving the rate of genetic gain for quantitative traits in sugarcane breeding (Yadav et al., 2020; Hayes et al., 2021; Islam et al., 2023).

Transgene is to introduce and integrate desired target genes into the genome of an organism so as to improve the original traits or endow them with desirable traits. Successful transduction of herbicide-tolerant *bar* gene, mosaic virus resistant *CP* and *P1* genes, and insect-resistant *bt* gene has opened up a vast field of genetic engineering in sugarcane, in which ‘CTC20BT’ and ‘CTC9001BT’ have been commercially grown in Brazil (Cheavegatti-Gianotto et al., 2019). Recently, genome editing especially CRISPR is emerging as a revolutionary technology in life sciences, which can realize targeted editing and modification to improve crop varieties according to breeding needs (Liu et al., 2022; Nerkar et al., 2022; Adeel and Jones, 2024). With the goal of high yield, high sugar and low fiber, a technical route for genome editing has now been established in sugarcane (Jung and Altpeter, 2016; Rahman et al., 2019; Laksana et al., 2024). The rapid development of high-throughput genomics technologies has brought life sciences into the era of big data, and omics-based interdisciplinarity is accelerating precision crop breeding (Bohra et al., 2020; Shen et al., 2022).

Surprisingly, the high polyploid and complex nature of the sugarcane genome make it difficult to breed new varieties. The genome has different ploidy levels and allele dosages (Batista et al., 2021), and it has also been a huge challenge to appropriately read the sugarcane DNA and distinguish among the different genotypic classes, mainly those related to heterozygous individuals (Garcia et al., 2013). In this sense, the majority of genotypic data, obtained for sugarcane to date, does not consider its ploidy levels, allele dosages and, therefore, does not provide a reliable genetic information about its genome (Yadav et al., 2023). At the same time, the statistical approaches are not fully developed to take into account the genotypic data obtained from the complex context of sugarcane, including the methods for GWAS, GS, and other approaches related to MAS (Yadav et al., 2020; Batista et al., 2021; Mahadevaiah et al., 2021; Jackson et al., 2022). Actually, for a long time, the sugarcane genome is mostly/still read and considered as diploid species, and the efficient use of these approaches is far from being a reality, although important progress has been made in the last years, and there is still a long way to realize molecular breeding for sugarcane.

### Breeding 4.0

**Figure 2:**
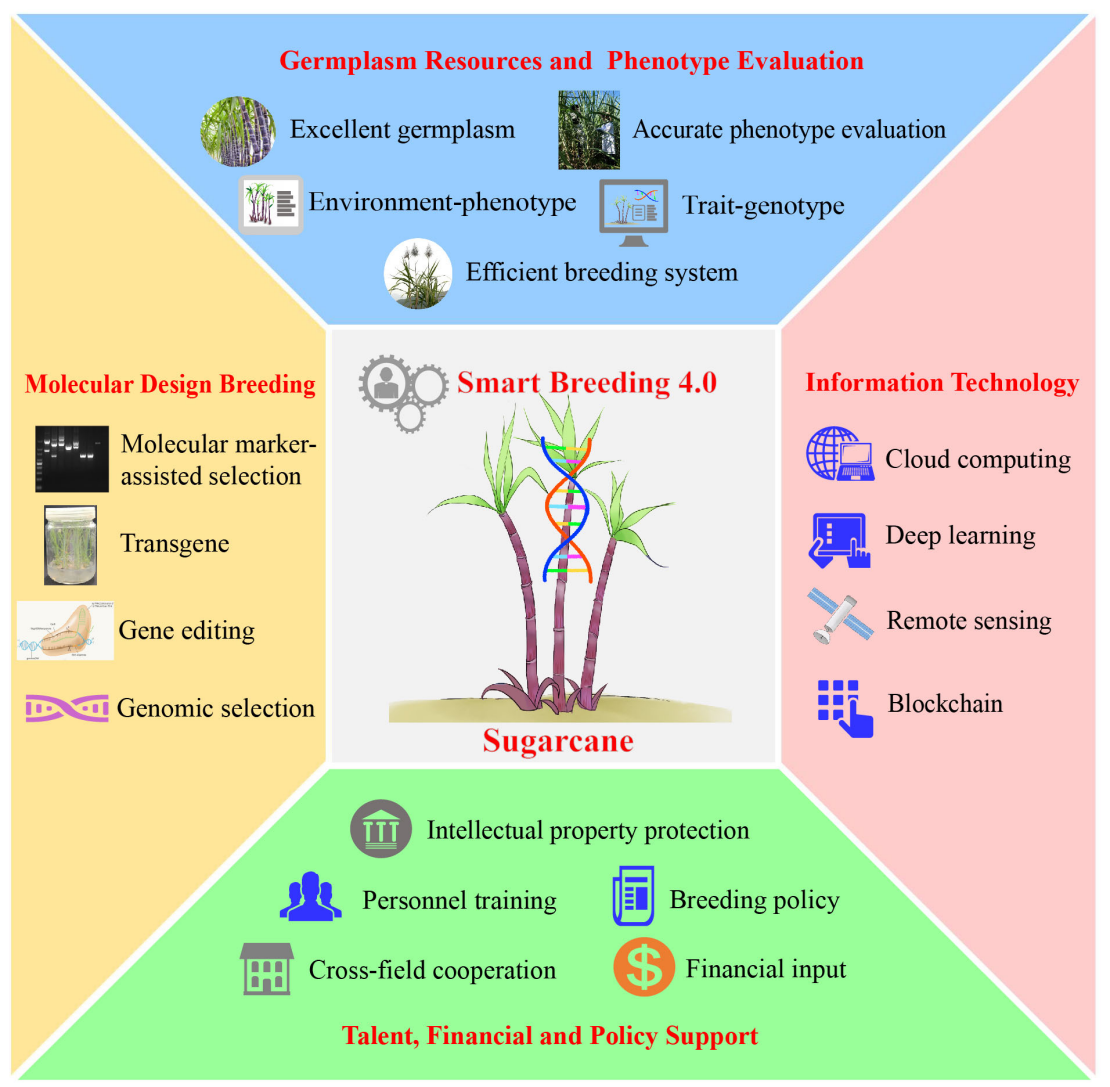
Framework for sugarcane breeding 4.0.

Smart breeding (4.0) is an emerging paradigm with combines genotype- and phenotype-based technologies, that deeply integrates modern biotechnology and information technology to achieve faster, better and more efficient breeding of new crop varieties (Chandra et al., 2024). According to the theoretical basis and technical means, breeding 4.0 can be divided into two main modes: The first is intelligent hybrid breeding, which uses MAS or GWAS strategies to aggregate exogenous/endogenous the key regulating genes into the target germplasm according to the breeding objectives. High-throughput phenotyping combined with remote sensing and deep learning can measure numerous traits with unprecedented spatial and temporal resolution, and quickly and accurately identify new germplasm or varieties combining many desirable traits (Mostafizur Rahman Komol et al., 2023; Kumarasiri et al., 2024; Santhrupth and Devaraj Verma, 2024). The second is intelligent biological breeding, which uses artificial intelligence to design superior allelic variants and genomic elements, and then uses transgenic approach and gene editing to write into the genome and precisely improve the target traits. The essence is the diversified integration of artificial intelligence such as big data and deep learning and biotechnologies such as gene editing and synthetic biology to achieve intelligent upgrading of biological breeding, which will substantially improve the utilization rate of germplasm resources (Rizzo et al., 2023; Yan and Wang, 2023). It is anticipated that smart breeding can provide an optimized way to address the challenges of difficult phenotypic identification, long breeding cycles and heavy workload in sugarcane breeding (**Figure 2**).

## Current status and challenges

### Current status for sugarcane breeding

Nowadays, there is a shortage of superior varieties with outstanding performance in various agronomic traits in sugarcane production. According to ISSCT, there are 56 countries with sugarcane breeding institutions in the world, among which Brazil, India, China, the United States, and Thailand have a perfect sugarcane breeding system and have already bred a number of elite varieties through hybridization or self-crossing (Zhou et al., 2023; Azim et al., 2024), such as the ‘RB’, ‘SP’ and ‘CTC’ series in Brazil, the ‘KK’, ‘K’, ‘LK’ and ‘UT’ series in Thailand, the ‘Co’ series of maincrop varieties in India, and the ‘GT’, ‘YT’ and ‘YZ’ series in China (Hu et al., 2021). These dominant varieties have given a strong impetus to the improvement of global average sugarcane yields and sugar per unit. According to FAO statistics on sugarcane, from 1961 to 2021, the unit production increased from 50.3 t/ha to 70.6 t/ha globally, with an increase of 20.3 t/ha and 40.4%; and the sugar content increased from only 9.1% to 14.0%. Moreover, a number of energy and sugar-energy sugarcane varieties have been selected, such as ‘US67-22-2’ and ‘IA313’ in the United States, ‘SP71-6163’, ‘SP76-1143’, ‘SP94-115’, ‘RB72-454’, and ‘RB85-5536’ in Brazil (Chen et al., 2011; de Souza Barbosa et al., 2020). However, existing sugarcane varieties are mainly bred from ‘POJ2878’ or its progenies, such as ‘F134’, ‘CP49-50’ and ‘NCo310’. Due to the similarity in parentage, the varieties have weakened disease and stress resistance, and their lodging and adaptability have deteriorated, thus limiting the sustained improvement in the sugarcane productivity (Chen et al., 2011; Zhao et al., 2022).

Collection, utilization, and innovation of germplasm resources are the source and key to breakthrough in sugarcane breeding, which need to be developed urgently (Huang et al., 2023). The Indian Institute of Sugarcane Breeding (Kannur) and the USDA Sugarcane Research Institute (Miami) are the two centers of sugarcane germplasm resources in the world, with 3,377 (Chandran et al., 2023) and 749 (Hale et al., 2022) germplasms, respectively. In the past 30 years, China’s National Germplasm Repository of Sugarcane (Kaiyuan) has collected and preserved 5,962 resources of six genera and 15 species from 39 major sugarcane-planting countries and the region of sugarcane germplasm resource origin centers (November 26th, 2023), and now it is the largest with the most complete genus and great diversity of preserved sugarcane germplasm resources in the world. In recent years, in order to expand the pedigree of sugarcane, Sugarcane Research Institute of Yunnan Academy of Agricultural Sciences in China has constructed an artificial photoperiod-induced hybridization technology system suitable for the Kaiyuan (location: 23°71’N, 103°26’E; altitude: 1,050 m), which overcomes the technical difficulties of distant hybridization between sugarcane genera, and successfully utilized *S. officinarum*, original cultivation varieties, and *S. arundinaceum* Retz. or *Erianthus rockii* Keng. for germplasm innovation.

### Challenges for sugarcane breeding

We have already entered into the early stage of Breeding 4.0 in rice and corn after years of development, however for sugarcane breeding, it is still at the stage of transition from conventional breeding (Breeding 2.0) to molecular breeding (Breeding 3.0) or their combination, largely lagging behind the main crops (Cursi et al., 2022a; Ram et al., 2022; Afzal et al., 2023; Azim et al., 2024; Qi et al., 2024). The major challenges in sugarcane breeding globally today are: (1) The narrow genetic base of modern varieties, with 78-80% of their genomes derived from *S. officinarum* and 10-20% from *S. spontaneum*, resulting in a serious lack of genetic variation in breeding populations, and limiting the effectiveness of varietal improvement (Piperidis et al., 2010). Besides, special attention should be paid on the potential of two strategies, recurrent GS and reciprocal recurrent GS, to increase the long-term genetic gain for complex quantitative traits in sugarcane breeding (Yadav et al., 2020). (2) Despite the current high number of bio-breeding studies, only Brazilian stem borer-resistant transgenic sugarcane has been used for production so far (Cheavegatti-Gianotto et al., 2019); The novel strategies of sequencing and genotyping for polyploids, especially sugarcane, as well as the development of specific statistical models to analyze the data, are an urgent demand for sugarcane breeding (Batista et al., 2021). (3) The development of accurate phenotype identification techniques for germplasm resources has lagged behind. (4) Existing informatization solutions have deficiencies in scale, performance, cost, and scalability, making it difficult to meet the needs of smart breeding.

## Conclusions and future prospects

Improvement and innovation of crop varieties is the top priority for securing the needs of human life and promoting social development. In the face of cross-pressures such as population explosion, decrease in available arable land, and increase in biotic and abiotic stresses, as a major sugar and energy crop, the varietal renewal of sugarcane will play a greater role in ensuring sugar supply. In the future, sugarcane breeding will focus more on ecologically viable, environmentally friendly and resource matching. It is thus suggested that we should continue to explore in depth the genetic characteristics of agronomic traits such as yield, sugar, and resistance, as well as amenability to mechanization. Special attention should be paid to the collection of wild germplasm resources and the innovation of germplasm parents. Combining with the means of transgene, gene editing, and synthetic biology, the innovation of technology and methodology can be strengthened for sugarcane breeding. We hope that in the near future, all those efforts can advance towards the higher goal of sugarcane breeding, in brief as high-yield, high-sucrose, stress resistance, and suitable for mechanization.

Smart breeding provides a good opportunity for varietal innovation in highly polyploid crops such as sugarcane. Grasping the current situation, it is important and necessary to build a sugarcane breeding 4.0 system adapted to the new era – “germplasm resources + biotechnology + information technology + talents + policies”. On the basis of in-depth exploration of the germplasm resources and genes in the “*Saccharum* complex”, we will continue to enrich and broaden the genetic foundation of sugarcane varieties through the combination of natural and artificial mutations, so as to cultivate new varieties with innovative and breakthrough features. Moreover, the process of digitizing sugarcane germplasm resources will be accelerated, and an integrated “germplasm conservation – variety breeding – seedling production” platform will be established to improve and precipitate the efficiency and accuracy of breeding. In summary, in the era of Breeding 4.0, it is promising to make significant breakthroughs in the field of sugarcane breeding, which should provide more elite varieties for the sustainable development of sugar industry.

## Data availability statement

The original contributions presented in the study are included in the article/supplementary material. Further inquiries can be directed to the corresponding author.

## Author contributions

GL: Formal Analysis, Methodology, Software, Visualization, Writing – original draft. PL: Visualization, Writing – review & editing. QW: Visualization, Writing – review & editing. SZ: Data curation, Writing – review & editing. PZ: Data curation, Writing – review & editing. YZ: Writing – review & editing, Conceptualization, Funding acquisition, Project administration, Resources, Supervision. YQ: Conceptualization, Funding acquisition, Project administration, Resources, Supervision, Writing – review & editing.
